# Impact of body mass index and socio-economic status on motor development in children and adolescents

**DOI:** 10.1007/s00431-021-03945-z

**Published:** 2021-01-28

**Authors:** Tanja H. Kakebeeke, Aziz Chaouch, Jon Caflisch, Elisa Knaier, Valentin Rousson, Oskar G. Jenni

**Affiliations:** 1 Child Development Center, University Children’s Hospital Zurich, Steinwiesstrasse 75, CH-8032 Zurich, Switzerland; 2 Division of Biostatistics, Center for Primary Care and Public Health (Unisanté), University of Lausanne, Lausanne, Switzerland; 3 Children’s Research Center, University Children’s Hospital Zurich, Zurich, Switzerland; 4 University of Zurich, Zurich, Switzerland

**Keywords:** Body mass index, Gender, Motor performance, Socio-economic status, Typical motor development

## Abstract

This study assessed the impact of body mass index (BMI) and socioeconomic status (SES) on the Zurich Neuromotor Assessment, second version (ZNA-2), a battery of tests of motor development in typically developing children between 3 and 18 years of age. BMI measurements and international socio-economic index data were taken from the normative sample of the ZNA-2 for 321 children (158 boys, 163 girls) with a median age of 9.3 years. The age- and gender-adjusted motor performance of these children was quantified and grouped into five components: fine, pure, and gross motor tasks, static balance, and contralateral associated movements. A total score was also calculated. The associations of BMI and SES with the motor scores contribute to less than 5.1% of the total variance.

*Conclusion*: The ZNA-2 for motor development is suitable for measuring motor abilities’ development as it is largely independent of the BMI of the child and the SES of the family.

**Table:** 

**What is Known:** • *Typical motor development, as measured with the Zurich Neuromotor Assessment, second edition (ZNA-2), is strongly dependent on age and gender.* • *The ZNA-2 focusses on motor performance, motor quality and simple motor skills.*	
**What is New:** • *Higher socio-economic status (SES) is associated with slightly better motor performance as measured by the ZNA-2 total score.* • *In the ZNA-2 less than 5.1% of the variability in motor performance is attributable to the combined effect of body mass index and SES.*

**What is Known:**

• *Typical motor development, as measured with the Zurich Neuromotor Assessment, second edition (ZNA-2), is strongly dependent on age and gender.*

• *The ZNA-2 focusses on motor performance, motor quality and simple motor skills.*

**What is New:**

• *Higher socio-economic status (SES) is associated with slightly better motor performance as measured by the ZNA-2 total score.*

• *In the ZNA-2 less than 5.1% of the variability in motor performance is attributable to the combined effect of body mass index and SES.*

## Introduction

The theory of changes in motor development has broadened from a maturational perspective to an ecological one [15]. This ecological perspective recognizes that both the body and the environment in which that body develops change and interact in a manner that can be difficult to predict [15]. Thus, when a clinician is interested in motor development at a purely neurological level, the ecological perspective obliges them to incorporate factors independent of age that may impact the motor proficiency of the child.

Indeed, not all changes in motor performance constitute development [15]. For instance, children who are heavier are morphologically constrained by their weight [2] and not developmentally delayed when their performance on skipping tasks is below average. The negative effect on motor performance of physical factors such as having too much weight is well established: children who are heavier are disadvantaged in antigravity motor skills [1, 2, 7, 12, 14, 24, 26, 27, 31, 34]. Conversely, children who play outside for more than 3 h per day—as recommended by the World Health Organization (WHO)—may have motor skills superior to those with a lower level of physical activity [1, 16, 25, 43]. Children who frequently play with balls may become skilled performers, but this progress is more a consequence of experience and practice than of development. We should primarily study the change in motor performance over time (which is development) rather than studying the impact of movement experience and weight on motor development. This implies that when we want to study motor performance and development, we have to consider the associations with the child’s body weight and environment.

The Zurich Neuromotor Assessment (ZNA [23]), its updated version (the ZNA-2 [18]) and the shortened one (the ZNA-Q [17]) are motor tests that describe the developmental course of motor performance and associated movements from childhood to adulthood. One feature of the ZNA-2 is that the tasks in this assessment are as gender- and culture-neutral as possible. It is known that test items that favour specific genders or cultures should not be part of a motor test focusing on performance [14, 28]. For instance, boys are usually better in all exercises with balls [1], as they practise this more; consequently, these exercises are not suitable for comparing motor performance between boys and girls. Likewise, competence in using pencils below age 5 is highly dependent on parental and childcare support. For this reason, tasks involving drawing are not suitable for investigating fine motor skills in children. In the ZNA-2, boys and girls are studied separately, as boys and girls have different levels of physical activity [8, 13, 39] and body compositions [3, 19, 20, 28, 38], which vary over age [3, 42].

Socioeconomic status (SES) is known to have an impact on motor performance and also requires consideration [6, 11, 14, 21, 30, 32, 40]. A child is embedded in an environment, and this environment may encourage more or less physical exercise and/or play. Motor development in a poor household with few opportunities may therefore lead to a child’s developing lower skills [1, 6, 14, 21, 30, 32, 40].

In the ZNA-2, gender differences were observed in many tasks [18]. Nonetheless, no empirical evidence is yet available about the effect of a biological factor such as BMI or an environmental factor such as SES on motor performance as evaluated with the ZNA-2. Roscoe et al. [35] investigated the influence of fundamental motor skills (including hopping, skipping and jumping) on physical activity levels and weight status in preschool children of low SES. Remarkable was that in this study no association was found between level of motor performance, physical activity, and weight status of the children. The results were explained by the fact that the effects of physical activity and weight on the functional motor skills were not yet fully established and therefore could not be linked to the level of motor performance of the children [35]. As this study was done only on children of 3–4 years old, it is interesting to know whether this effect is detectable in children older than 4 years old.

In this study, we aim to investigate the magnitude of the effects of BMI and SES on motor performance in typically developing children from 3 to 18 years old. Quantifying such effects is important when considering typical motor development. Only by studying this association in typically developing children and adolescents may we disentangle the associations of non-motor-dependent factors such as BMI and SES from the development of motor performance.

## Methods

### Participants

From the original 616 children and adolescents of the ZNA-2, a normative sample of typically developing children and adolescents [18] of 321 participants (158 males, 163 females) with complete measurements for height, weight and parental occupational status were gathered. From June 2015 until February 2017, a representative sample of the general population living in the greater Zurich area was tested. Children were tested in their own day-care centres and schools in a separate room. Children with evident medical or behavioural conditions were not asked to participate. The children and adolescents’ ages ranged from 3 years and 3 months to 17 years and 11 months with a median age of 9 years and 4 months; the sample contained 256 children (≤ 12 years) and 65 adolescents (> 12 years). Participants were enrolled from six daycare centers, five kindergartens, and 17 primary and secondary schools covering the richer and poorer areas of the urban area. Their motor proficiency was assessed using the ZNA-2. The study was approved by the local ethical committee (KEK-ZH-Nr StV-40/07) and was performed in accordance with the Declaration of Helsinki. Details of the study design have been described previously [18].

### Measurements

#### Zurich Neuromotor Assessment-2 (ZNA-2)

The ZNA-2 allows continuous measurement for the entire age range without changing the test items. Motor proficiency is assessed with 14 tasks grouped into five components: fine motor tasks (FM), pure motor tasks (PM), static balance (SB), gross motor tasks (GM), and contralateral associated movements (CAMs). The FM component entails a pegboard task, turning bolts, and stringing beads. The PM component consists of repetitive movements of fingers, hand and foot, alternating movements of hand and foot, and sequential finger movements. SB consists of standing on one leg with eyes open and with eyes closed. The GM component entails jumping sideways, a chair-rise test, and standing long jump. CAMs are scored from video recordings for 5 out of the 14 tasks. They are treated as a separate component that measures the quality of movement during the fine and pure motor tasks. CAM scores capture the frequency (i.e., during the whole movement or only part of it) and amplitude of the movement on the contralateral side of the limb being tested. Besides components, the ZNA-2 also provides a global measure of motor proficiency using a total score. The total score (TS) summarizes information collected over all five components.

#### Body mass index (BMI)

All subjects were tested in the childcare centre or at school. Height was measured with a stadiometer to the nearest 0.1 cm, and weight was measured using a scale (Seca, Basel, Switzerland). Both measures were evaluated with the subject barefoot and not immediately after a heavy meal. BMI was calculated as weight divided by height squared (kg/m^2^). Over- and underweight was defined according to the definitions of the WHO [42].

#### Socio-economic status (SES)

The International Socio-Economic Index (ISEI) was calculated by coding the occupational status of both parents [9]. The highest ISEI of the two parents was selected and used for the analysis as a proxy for the SES of the child.

### Procedure

Each child was examined individually to evaluate motor performance by a professional therapist or human movement scientist trained in the ZNA-2. In total, five people were trained for the ZNA-2 assessment; all testing and scoring were supervised by the most experienced ZNA testers, TK and JC. Details of the motor testing procedure and the psychometric properties of the ZNA-2 can be read elsewhere [18]. After motor testing, all participants received a questionnaire for the parents requesting information on several topics including SES. These questionnaires were returned in sealed envelopes. Each letter contained an identification code instead of the participant’s name so as to blind the study investigators entering the information into the database.

### Statistical methods

In order to remove the impact of age and gender from the analysis, BMI values were converted into *z*-scores using BMI-for-age standards from WHO Child Growth Standards (3–5 years) and Growth Reference (5–19 years) [5]. *Z*-scores of BMI were restricted to the interval [− 3; +3] to prevent extremes from driving the analysis. The raw ISEI was used for the analysis. When appropriate and to improve the interpretability of results, the ISEI was standardized around its median (65) and divided by 30 points (rounded interquartile range).

Motor performance was measured as the time needed by the child to complete the task, or by measuring the distance a child was able to jump. The frequency and amplitude of CAMs were combined into an index called the intensity of CAMs [10]. This index, essentially the square root of the product of frequency and amplitude, provides information on movement quality. It can take 24 possible values, ranging from 0 (no CAMs) to 5 (maximal intensity of CAMs), and is approximately normally distributed within these bounds in the population of typically developing children. Norms for motor development were then used to calculate standard deviation scores (SDSs) for motor performance across items, components, and the total score of the ZNA-2 [18]. Such norms describe the typical evolution of the time performance and intensity of CAMs as a function of age and gender. Similar to a *z*-score, an SDS is a quantitative measure of age- and gender-adjusted motor performance, with positive SDSs indicating above-average performance and negative SDSs indicating below-average performance. By construction, SDSs are approximately normally distributed with a mean of zero and a variance of one in the population of healthy children. As for BMI *z*-scores, motor SDSs were restricted to the interval [− 3; + 3] to limit the influence of extremes. SDSs missing at random or missing due to the inability of the child to perform were imputed using a specific procedure for multiple imputations with chained equations (see statistical appendix for details). Fifty complete datasets were generated using this procedure in order to reconstruct the full variability of SDSs.

Linear mixed effects models were used to quantify the association of BMI *z*-scores and the ISEI with motor SDSs across the five components and the total score defined in the ZNA-2. Cluster effects due to children being nested within schools were incorporated as random intercepts. The effects of BMI *z*-scores and ISEI were flexibly modelled using fractional polynomials of degree 2 to capture nonlinear trends [36]. Models were fitted successively to each of the 50 complete datasets constructed using the imputation procedure, and regression coefficients, standard errors and so forth were then pooled using Rubin’s rules to enable inference following repeated imputations [37]. The effect of BMI *z*-scores and of ISEI on motor performance was estimated and the corresponding 95% confidence interval was reported. For linear trends, the *p* value corresponding to a test for the nullity of the slope of the regression line was reported. Nonlinear trends were tested using a likelihood ratio test (LRT) with a procedure adapted to handle multiple imputations [29]. A term representing the interaction between the two predictors was also tested using the same procedure. The level of statistical significance was set to 5%. The magnitude of the cluster effect was quantified using the intraclass correlation coefficient (ICC).

## Results

After adjusting for the cluster effect, BMI *z*-scores for the 321 children under study were neither associated with age (*p* = 0.549) nor ISEI (*p* = 0.397). However, on average, boys had a slightly larger BMI *z*-score (delta *z* = 0.26) than girls, with this difference being statistically significant (*p* = 0.023). The cluster effect for BMI *z*-scores was negligible, with variations between schools explaining less than 1/10,000^th^ of the total residual variance. The average ISEI was 59 points (SD = 20), which stands slightly above the Swiss average of 55 points (SD = 17) [33]. The median ISEI was 65 points with an interquartile range of 29 points. The ISEI distribution was bimodal with a main peak at higher values of the index (60–80) and a smaller peak at lower values (20–40). ISEI was not associated with age, gender, or BMI *z*-scores (*p* ≥ 0.156). However, unlike for BMI, the cluster effect for ISEI was substantial, with variations between schools explaining 39% of the total residual variance.

**Fig. 1:**
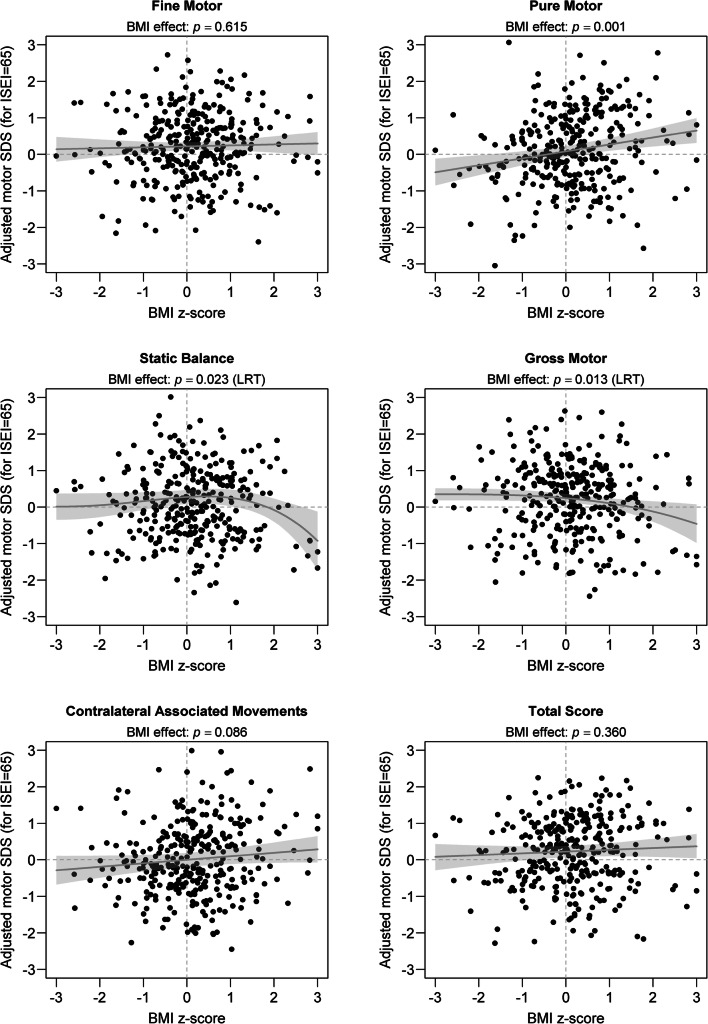
Adjusted motor SDS in ZNA-2 components and total score as a function of BMI *z*-scores. For illustrative purposes, the predicted difference between the actual ISEI and a median ISEI of 65 points is subtracted from each motor score. Dots refer to the adjusted motor scores, observed or imputed from the first complete dataset. The grey line represents predicted motor scores as calculated after pooling regression coefficients from the 50 complete datasets. The light grey area represents a 95% confidence interval around the regression line. For linear effects, the reported *p* values refer to testing for nullity of the slope of the regression line. The *p* values reported for nonlinear effects (Static Balance and Gross Motor) were obtained using a likelihood ratio test (LRT)

Table 1 presents means and standard deviations of motor SDSs for components and the total score in the ZNA-2 as calculated after pooling results from the 50 complete datasets with observed and imputed data. On average, motor SDSs were positive on all components and the total score of the ZNA-2. However, only the average SDSs of FM and GM were statistically different from zero after accounting for the cluster effect. In multivariate models, the effect of BMI *z*-scores on motor SDSs was statistically significant only in components PM, SB, and GM (*p* ≤ 0.023). Generally, BMI *z*-scores were linearly related to motor performance, with overweight children having on average slightly better motor scores than underweight children (see Fig. 1). Notable exceptions are SB and GM, in which a nonlinear effect of BMI *z*-scores on motor SDSs was identified (LRT: *p* = 0.023 and *p* = 0.013, respectively). For these two components, average motor scores remained relatively unaffected by BMI except for severely overweight children (BMI *z* > 2), whose motor scores dropped significantly (see Fig. 1).

**Table 1:** Mean and standard deviation (SD) of motor SDSs for ZNA-2 components and total score (results pooled over the 50 complete datasets used in multiple imputations)

ZNA-2 dimension	Mean	SD	*p* value*
Fine motor	0.18	0.95	0.002
Pure motor	0.10	1.01	0.194
Static balance	0.17	1.02	0.189
Gross motor	0.15	1.00	0.038
Contralateral associated movements	0.02	1.02	0.996
Total score	0.19	0.96	0.062

*The *p* value refers to testing for nullity of the mean SDS value while accounting for the clustered design. This is achieved by using a linear mixed effects model without any predictor while including schools as random intercepts to predict motor performance SDS on a given ZNA-2 component

**Fig. 2:**
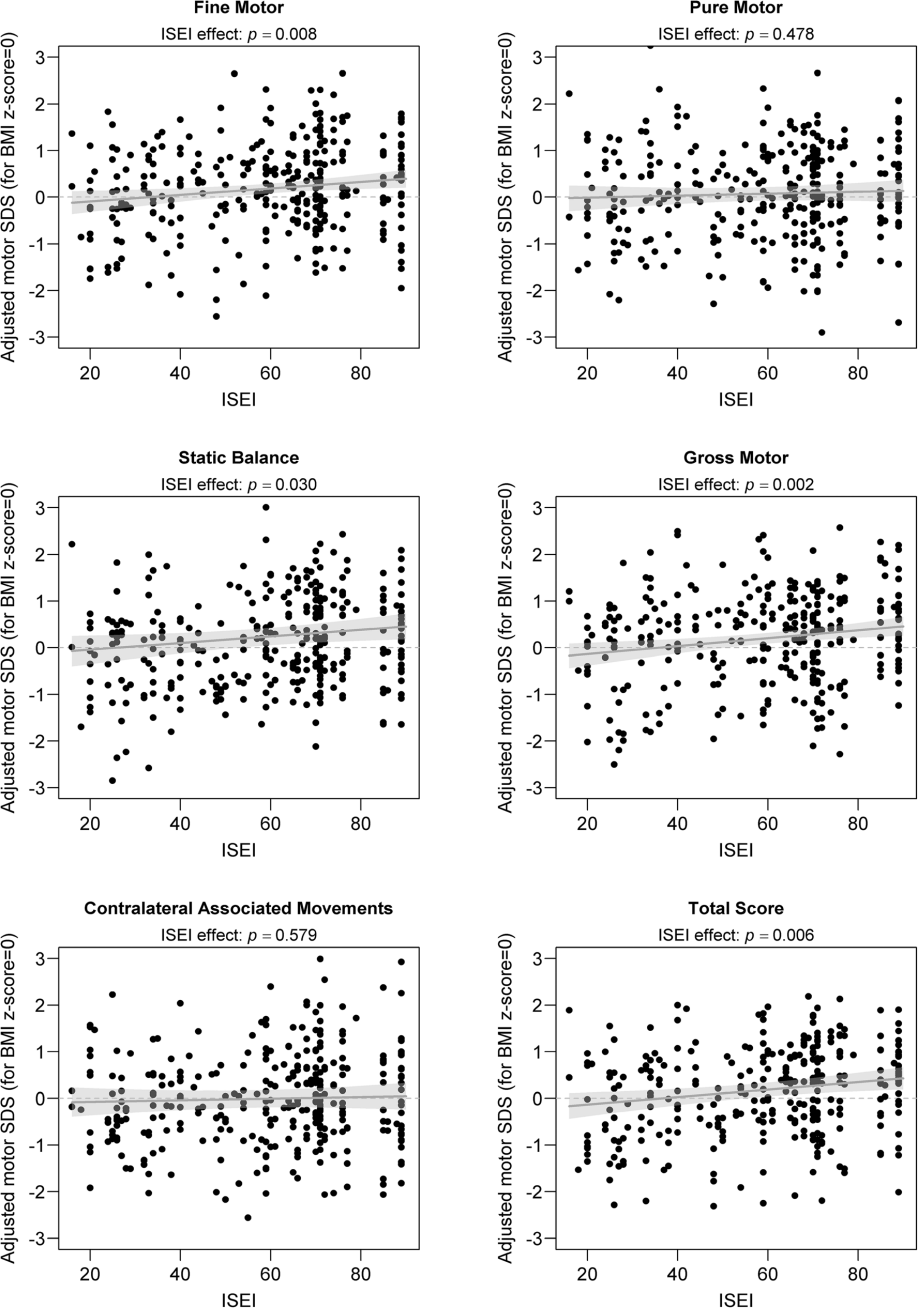
Adjusted motor SDS in ZNA-2 components and total score as a function of ISEI. For illustrative purposes, the predicted difference between the actual BMI *z*-score and a *z*-score of zero is subtracted from each motor score. Dots refer to the adjusted motor scores, observed or imputed from the first complete dataset. The grey line represents predicted motor scores as calculated after pooling regression coefficients from the 50 complete datasets. The light grey area represents a 95% confidence interval around the regression line. The reported *p* values refer to testing for nullity of the slope of the regression line

With the exception of PM and AM, the ISEI was consistently associated with motor scores, with children from higher socio-economic backgrounds performing slightly better than those from lower socio-economic backgrounds (*p* ≤ 0.030). Unlike for BMI, where a nonlinear effect could be identified in the SB and GM components, the association between ISEI and motor scores was constantly modelled as linear for all components and the total score of the ZNA-2 (see Fig. 2). An interaction term between ISEI and BMI *z*-scores was never found to be statistically significant (LRT: *p* ≥ 0.195) and was thus not considered in final models.

The magnitude of the cluster effect on motor performance was generally small. The ICC on the total score TS was 0.037, suggesting that after adjusting for the combined effect of BMI and ISEI, differences in average motor performance between schools only account for 3.7% of the total residual variance. The largest cluster effect on motor performance was found for the component CAM, with an ICC of 0.08.

Although sometimes statistically significant, the magnitude of the association between the two correlates investigated and motor scores always remained relatively weak. For example, when comparing children with a low BMI (situated at quantile 25% or *z* = − 0.67) and children with a high BMI (situated at quantile 75% or *z* = + 0.67), having the same age, gender and SES, we will observe an average difference of at most 0.26 SDS [95% CI 0.11–0.40] (in PM, see Table 2) regarding neuromotor performance. This corresponds to a weak effect according to the well-known classification of Cohen’s standardized mean difference [4]. Similarly, the largest predicted linear change in motor SDS, associated with an increase of 30 points on the ISEI scale (corresponding approximately to the interquartile range of ISEI in the sample) was 0.25 SDS [95% CI 0.09–0.41] (in GM, see Table 3). Another way to quantify the magnitude of these weak associations is to calculate the partial correlation coefficient between motor performance and each predictor: the correlation after adjusting both motor performance and the predictor of interest for the effect of the other predictor and school effects. The largest absolute partial correlation between motor performance and BMI *z*-scores was only 0.19 (in PM) while the largest absolute partial correlation between motor performance and ISEI was even smaller at 0.13 (in TS). Larger deleterious effects of BMI on motor performance may be seen for SB and GM but only in severely overweight children (see Fig. 1). As a post hoc analysis, we formally tested for the presence of an interaction term between age (categorized into children, age ≤ 12 years, and adolescents, age > 12 years) and BMI *z*-scores in all motor dimensions of the ZNA-2. This interaction term was not supported by the data (LRT: *p* ≥ 0.515) in any motor components except PM, where the positive effect of BMI *z*-scores disappeared in adolescents (LRT: *p* = 0.036). Overall, in multivariate models, BMI *z*-scores and ISEI together accounted for no more than 5.1% of the total variance in motor SDSs. In univariate models, the largest proportion of variance in motor SDSs explained by BMI *z*-scores and ISEI was only 3.4% (in PM) and 3.8% (in TS), respectively.

**Table 2:** Regression coefficients and 95% confidence intervals (CI) for the linear effect of BMI z-scores on motor performance for ZNA-2 components and the total score, after controlling for ISEI and cluster effects (results pooled over the 50 complete datasets used in multiple imputations). The reported estimate corresponds to an increase of 1.35 points on the *z*-score scale for BMI (corresponding to the interquartile range of a standard normal distribution)

ZNA-2 dimension	Estimate	95% CI	*p* value
Fine motor	0.04	[− 0.10; 0.17]	0.615
Pure motor	0.26	[0.11; 0.40]	0.001
Static balance	Nonlinear effect (see Fig. 1)		*0.023
Gross motor	Nonlinear effect (see Fig. 1)		*0.013
Contralateral associated movements	0.13	[− 0.02; 0.28]	0.086
Total score	0.07	[− 0.07;0.20]	0.360

*The *p* values reported for nonlinear effects were obtained using a likelihood ratio test (LRT)

**Table 3:** Regression coefficients and 95% confidence intervals (CI) for the linear effect of ISEI on motor performance for ZNA-2 components and the total score, after controlling for BMI *z*-scores and cluster effects (results pooled over the 50 complete datasets used in multiple imputations). The reported estimate corresponds to an increase of 30 points on the ISEI scale (corresponding to the interquartile range in the sample)

ZNA-2 dimension	Estimate	95% CI	*p* value
Fine motor	0.21	[0.06; 0.36]	0.008
Pure motor	0.06	[− 0.11; 0.24]	0.478
Static balance	0.21	[0.02; 0.40]	0.030
Gross motor	0.25	[0.09; 0.41]	0.002
Contralateral associated movements	0.05	[− 0.13; 0.24]	0.579
Total score	0.24	[0.07; 0.41]	0.006

## Discussion

Ideally, a test set designed to measure motor development, coordination, and impairment (such as for children with developmental coordination disorder, prematurity, or special motor problems) should be as independent as possible from confounding, non-motor, and environmentally driven effects. This study has shown that the association of BMI and SES with motor development in children aged 3 to 18 years old as measured by the ZNA-2 is very low.

ISEI was found to have a small association with motor performance in the FM, GM and total score of the ZNA-2, although the magnitude of ISEI’s effect was weak (max. 3.8% of explained variance in motor SDSs) and not statistically significant in SB, PM and CAM. The association was consistent, such that higher ISEIs were associated with better motor performance. Children from higher SES backgrounds are known to play sports more frequently [1, 6, 11, 28, 30, 43]. However, although sometimes statistically significant, these associations remain clinically irrelevant in our study due to the low proportion of explained variance (≤ 3.8%).

The effect of BMI was even weaker (max. 3.4% of explained variance in motor SDS) but differed across the five components. The associations of BMI with FM and CAM were not significant. BMI showed a significantly positive association with PM and a negative association with SB and GM. Greater weight indeed makes performing activities against gravity more difficult. The negative association between BMI and SB has also been found elsewhere [31, 34]. For both SB and GM, a drop in performance was seen in children with BMI *z*-values of over 2.

The positive association between BMI and PM is more difficult to explain. BMI is a crude measure of body composition and does not indicate whether a child has more muscle mass (fat free) or more fat mass. The specific weight of muscle is higher than that of fat. It might be that among those children with high BMIs, some had relatively more muscle, leading to better performance in the PM tasks. This effect was also described in an earlier work on children between the ages of 3 and 5, in which children with high BMIs were also better in certain tasks [19]. Age may also be a confounding variable for the effect of BMI *z*-scores on PM in our study. Indeed, fat-free mass is known to increase with age, with adolescents typically having more fat-free mass than younger children [3, 22, 41]. The post hoc analysis confirmed that the positive effect of BMI on PM found in younger children (≤ 12 years) disappeared in adolescents. This suggests that the positive association between BMI *z*-scores and PM may be driven by changes in fat-free mass over age. Finally, it should be noted that we did not have many children with high BMIs in this cohort.

One limitation of our study is that the 321 children included in the analysis do not form a random sample of children from the ZNA-2 normative sample. Instead, they were selected based on the availability of information on their parents’ occupations to enable an ISEI to be calculated. We suspect that this subsample may thus include a larger proportion of children with higher SES than the complete ZNA-2 normative sample. Indeed, parents with lower SES may be less likely to report their occupations than parents with higher SES. This suspicion is supported by two observations. First, both the average ISEI (59) and the median ISEI (65) in our sample are slightly higher than the Swiss average (55) [33]; the ISEI distribution is bimodal with a main peak at higher ISEI (60–80). Secondly, we observe that the average motor SDSs of the selected children are slightly higher than zero across all ZNA-2 dimensions, yet not always significantly so (see Table 1). The selected children may thus have performed slightly better than the typically developing children of the whole ZNA-2 normative sample. However, this slight shift towards better motor performance is readily compatible with the ISEI effect depicted on FM, GM, and the total score of the ZNA-2.

Note also that we did not observe any significant effect of ISEI on PM or CAMs. This further explains why the average motor SDSs for these two components are not statistically different from zero in Table 1 and supports our hypothesis that PM and CAMs are independent of SES. One possible consequence of this selection process is that the SES effects reported in this study are likely to be slightly underestimated. Nevertheless, we do not believe this to be a strong limitation of our study, for two reasons. First, although children with lower SES may be under-represented in our sample, the whole range of ISEI was observed. Secondly, the proportion of explained variance in motor SDS that is attributable to the combined effect of BMI and ISEI was only 5.1% at most. It is quite possible that this number is slightly underestimated, but we do not believe the proportion of explained variance due to BMI and ISEI would reach any relevance for clinical practice in the absence of the selection process. Therefore, we remain confident that the effects of BMI and SES can reasonably be neglected in clinical practice as was also seen in a study on 3- to 4-year-olds [35].

The ZNA-2 enables the continuous assessment of motor performance in children and adolescents from 3 to 18 years. It provides normative data for motor proficiency while accounting for gender differences. Our findings suggest that motor performance can be reliably quantified with the ZNA-2 without suffering from any strong effect of such non-motor factors as BMI or SES.

### Statistical appendix

#### Imputation of missing motor SDSs

For some items in the ZNA-2 (e.g. SB, CAMs), the range of motor performance that can actually be measured is bounded. For example, the time over which a child can stand on one leg is restricted to the interval 2–30 s. Times shorter than 2 seconds (floor limit) are difficult to measure reliably. On the other hand, allowing a child to stand on one leg for more than 30 s (ceiling limit) would make the whole testing procedure too time consuming in practice. Another example is the intensity of CAMs which is bounded by design between 0 (floor limit) and 5 (ceiling limit). Building on the approach of Gasser et al. [10], the ZNA-2 norms properly account for such peculiarities by considering that the true motor performance of a child who reaches, e.g. the ceiling limit in SB is right-censored at 30 s (i.e. his/her true exact performance is unknown, but it is known to be at least equal or larger than 30 s). Similarly, norms for CAMs assume that the true intensity of CAMs of a child, e.g. who reaches the ceiling limit, will be right-censored at 5, with his/her true intensity of CAMs being known to be equal or larger than 5 although such value cannot be measured in practice. The SDS of a child who reaches either the floor or ceiling limit is calculated approximately by considering the fraction of children of same age and sex whose motor performance lies below the floor limit or above the ceiling limit according to the norms (see [18] for more details). While such approximation is valuable to provide an estimate of the motor performance for those children reaching the floor or ceiling limit, it does have one drawback: all children of the same age and sex who reach, e.g. the ceiling limit, will get exactly the same SDS. When the proportion of children reaching that limit is large (e.g. when many young children reach the maximum intensity of CAMs), the SDS variability decreases drastically, which is undesirable if one is specifically interested in explaining some of the variability in motor performance with predictors such as BMI and/or SES. Consequently, for a child reaching the ceiling limit on, e.g. SB, we imputed a standard normal SDS value in the interval [Φ^−1^(*p*_*CL*_); 3] where *p*_*CL*_ is the percentile corresponding with the ceiling limit (30 s) in the norms, and Φ refers to the cumulative distribution function of the standard normal distribution. Similarly, a standard normal SDS value in the interval [− 3;Φ^−1^(*p*_*FL*_)] was imputed for a child reaching the floor limit, with *p*_*FL*_ referring to the percentile corresponding to the floor limit (2 s.) in the norms. SDSs missing at random were imputed in the interval [− 3;+3] using a standard normal distribution. The imputation procedure was repeated 50 times in the spirit of multiple imputations [37]. The imputation model for motor SDSs in any particular task included the following predictors: SDSs from all remaining ZNA-2 items, age, gender, BMI z-scores, ISEI and an interaction term between BMI z-scores and ISEI. Additionally, quadratic effects for age, BMI *z-*scores and ISEI were included in the imputation model to capture potential nonlinear trends. The SDS imputation was only carried out at the item level, with SDSs of components and the total score calculated from observed and/or imputed SDSs at the item level.

## Abbreviations

BMI: Body mass index
CAM: Contralateral associated movements
FM: Fine motor
GM: Gross motor
ICC: Intraclass correlation coefficient
ISEI: International Socio-Economic Index
LRT: Likelihood ratio test
M-ABC-2: Movement ABC (second version)
PM: Pure motor
SB: Static balance
SDS: Standard deviation score
SES: Socioeconomic status
TS: Total score
TGMD-3: Test of Gross Motor Development (third version)
WHO: World Health Organization
ZNA-2: Zurich Neuromotor Assessment (second version)
